# Multifaced Evidence of Hospital Performance in Pennsylvania

**DOI:** 10.3390/healthcare9060670

**Published:** 2021-06-04

**Authors:** Younhee Kim, Keon-Hyung Lee, Sung W. Choi

**Affiliations:** 1 School of Public Affairs, Pennsylvania State University Harrisburg, Middletown, PA 17050, USA; yzk46@psu.edu (Y.K.); sxc835@psu.edu (S.W.C.); 2 Askew School of Public Administration and Policy, Florida State University, Tallahassee, FL 32306, USA

**Keywords:** benchmark, data envelopment analysis, efficiency scores, Malmquist productivity indices, market competition, productivity changes

## Abstract

As health care costs and demands for health care services have been rising for decades in the United States, health care reforms have focused on increasing the performance of health care delivery. Competition has been considered as a mechanism to improve the quality of health care services and operational performance. Evidence on health care performance and market competition, however, has not sufficiently been reported to track its progress. The purpose of this study is twofold: First, we measure hospital performance over nine years, using the Malmquist Productivity Index. Second, we examine the impact of market competition on hospital efficiency in Pennsylvania, using a two-stage estimation procedure. The bootstrapped Malmquist productivity indices resulted in noticeable performance improvements. However, no steady performance trends were found during the course of nine years. In examining the impact of market competition, the bootstrapped panel Tobit analysis was applied after computing the efficiency scores with Data Envelopment Analysis. The results of the Tobit model found that hospitals run more efficiently in less competitive regions than in more competitive regions. The finding implies that hospitals underperforming in productivity growth should benchmark best practices of efficient hospitals to improve their productivity level. Another implication is that market competition would not be the best approach to effect the improvement of hospital efficiency in delivering health care services.

## 1. Introduction

The health care industry in the United States has faced increasing pressures in responding to expensive health care services and a shortage in the medical workforce. Maximizing health care performance in hospitals has been prioritized but the use of health care resources has not been relatively efficient to improve health care delivery [1]. The role of competition is perceived as an enabler that pushes health care systems to improve access to hospital services and reduce costs [2,3]. Although market competition is not a panacea to drive down challenging health care issues, competition-based health systems can assure the improvement of health care services and hospital management over time. However, it is evident that the relationship between market competition and hospital efficiency has been inconclusive and has had mixed results [3,4,5,6,7,8,9,10,11,12,13,14,15,16]. 

Studies have argued that hospitals that were exposed to more competitive market pressures were then enabled to lower hospital costs [15,17] and the length of patient stays [7]. With the market-based reform, hospitals also achieved a higher quality of care [7,18,19]. On the other hand, studies also found that a competitive environment did not lead to a higher level of efficiency [4,9,16] and the patient outcome [15]. The hospitals in the more competitive markets had higher costs than those in less competitive markets because they had amenity-based quality with expensive medical equipment, a fee-for-service reimbursement system [20], a third-party insurance system, and expanding services [21]. With these mixed results, the relationship between market competition and hospital efficiency should be carefully examined as hospitals may inefficiently allocate their resources when public hospitals have to compete predominantly with a large number of private hospitals [5]. Moreover, the competition effect can vary with hospital size [13,22].

A lack of consensus on how to measure the performance of hospitals’ health care services also contributes to the difficulty of predicting these relationships despite widespread interest in evaluating efficiency. Studies have argued that measurement in health care delivery has lagged and has been less routinized compared to other fields [10,23,24]. Health care inefficiency was often caused by inappropriate measures, methods, and units of analysis for service activities [25]. Data envelopment analysis (DEA) has been popular for estimating performance at different levels and unites of health care services [26]. DEA models offer conceptual clarity of performance assessment based on explicit purposes when using comparable multiple inputs and outputs metrics to calculate the relative efficiency of organizational unites.

Pennsylvania has remained relatively unknown for its level of hospital performance in health care services and the impact of its market competition on hospital performance as well. Chen et al. [27] analyzed the impact of the Great Recession on hospital efficiency in Pennsylvania and found that the efficiency of hospitals decreased by 2.43–3.07 percentage points during the period 2005–2012. Forty-three hospital mergers and acquisitions occurred among competing hospitals in Pennsylvania from 2000 to 2010, while its bordering states including New York, New Jersey, and Maryland had 17, 14, and 8 hospital mergers and acquisitions, respectively, in the same time period [28]. These mergers and acquisitions reduced competition for hospital services with the expectations of increasing hospital efficiency. It would be worthwhile to explore the condition of the performance trends during this radical transition.

The aim of this study is twofold. The first is to assess hospital performance in Pennsylvania over the period 2001–2009, using a bootstrapped Malmquist productivity index approach to track productivity changes with panel data. As the original DEA efficiency scores are not comparable across different periods [29,30], calculating a level of efficiency over time needs to control changes in the efficient frontier over time or include both years. The second is to examine the impact of market competition on hospital performance by using the two-stage producers. A bootstrapped DEA was first applied to estimate efficiency scores, and then Tobit analysis was conducted to test the relationship between market competition and hospital performance. The rest of the paper is divided as follows: we outline the data and methods; we present our results; and then we offer a discussion and conclusion.

## 2. Data and Methods

### 2.1. Data

This study used the panel data of general acute-care hospitals in Pennsylvania, collecting them from the American Hospital Association’s annual hospital surveys and the Center for Medicare and Medicaid Services’ Medicare cost reports of hospitals over the 2001–2009 period. While the original data included 178 general acute-care hospitals in 2001 to 153 in 2009, to construct a balanced dataset for the Malmquist technique, this study used 78 hospitals per year. The multiple imputation approach modeled by Honaker and King [31] was considered to hold hospitals with small missing values. However, the imputation method may generate outliers in the original data for DEA, which will result in relational changes in the original data. As a result, this study dropped hospitals with missing values over the nine years.

### 2.2. Model Specification

DEA has been the most prevalently applied method to measure efficiency in health care services [32]. This technique defines a hospital’s relative efficiency status compared with the benchmarked hospital group based on a ratio of the prices of the inputs and outputs. An efficiency score indicates how well an individual hospital performs compared to its benchmarked peer group. It can also use multiple inputs and outputs together to compute a hospital’s efficiency gap from the best practice group. DEA positions two scales of efficiency - input and output orientation [25,26,33]. Input orientation aims to minimize input usage at a given output level, while output orientation targets to maximize outputs at a given input level. The DEA approach assumes two distance functions by constant returns to scale (CRS), or variable returns to scale (VRS) [25]. CRS assumes that all inputs and outputs are proportionally increased. On the other hand, VRS assumes that inputs and outputs are disproportionally increased, which is more flexible than CRS.

DEA is typically constricted for one year, so efficiency scores cannot be compared to track its performance progress across years. Alternatively, this study conducted a bootstrapped Malmquist Productivity Index approach to estimate annual performance across the nine years. The Malmquist index method is the most commonly used method for measuring productivity changes during different years [34,35]. DEA is a data-centered approach, so it is unrestricted when specifying parsimonious functional forms or assumptions. As a non-parametric mathematical programming technique, DEA is convenient to apply but also could be vulnerable to sampling variation, measurement errors, and inferential validity. In minimizing the inherent drawbacks of original DEA techniques, this study applied a bootstrap specification to correct the efficiency measures’ bias and estimate the sampling variation of efficiency estimators. The bootstrap techniques were implemented to determine whether the estimates of specific distance functions are conditioned on the original data or the pseudo-data of inputs and outputs. This study adopted Löthgren and Tambour’s algorithm [33] to compute the bias-corrected Malmquist indices.

The bootstrapped Malmquist productivity index applied in this study is composed of efficiency change (EC) and technical change (TC) to compute the Total Factor Productivity (TFP) of a decision-making unit (DMU) between *t* and *t* + 1. EC measures the catch-up of a DMU towards the frontier in an output-oriented distance function, while TC indicates the shift in the frontier in two periods [36]. TFP reflects the degree to which DMU has taken advantage of both possibilities to present the productivity change between *t* and *t* + 1. If TFP is greater than 1, productivity growth happens between two periods. TFP = 1 indicates no productivity change, while TFP < 1 represents productivity decline. The Malmquist index = 1.1 is interpreted as a productivity growth of 10% compared to the previous period.

In examining the impact of market competition on hospital performance, this study proceeded with a two-stage approach with panel data. The traditional two-stage approach is used to estimate input-oriented or output-oriented efficiency scores in a first step and then regress these efficiency estimators over covariates as a second step using a bootstrapped panel Tobit model with a censoring point at zero. The bootstrapping method was performed to construct statistical proficiency with 2000 replications. As the U.S. economy grew on average by about 1% per annum from 2001 to 2009, health care expenditures and the health care workforce were also comparably increased. An opportunity to improve the performance of health care delivery should reveal output increases when inputs hold steady, so this study estimated an optimal solution of output-oriented efficiency scores (theta) through the DEA model. The DEA efficiency scores ranged from 0 to 1, in which a score of 1 represents a technically efficient level, and a score of less than 1 indicates an inefficient level.

### 2.3. Measures

A selection of inputs and outputs to assess hospital performance should be based on a correlational and judgmental relationship. It is critical to use appropriate inputs in the process of producing high-value hospital system outputs. In health care services, inputs are typically a series of cost and labor resources, and outputs are the services delivered. However, outputs are relatively challenging to operationalize related to health gains. Previous studies at the hospital level used full-time equivalent personnel and beds as input measures [12,37,38,39]. As practical measurement instruments, inpatients, outpatient visits, and surgical operations were considered [12,37,40]. While no adequate quantity of an input and output combination has been advised, the use of multiple inputs and outputs are suggested to prevent an arbitrary or selective decision, as opposed to using a single input and output combination.

This study used three inputs and three outputs commonly applied to compute health care efficiency at the hospital level corresponding to our study objectives. These are presented in Table 1. As a proxy for a financial input, beds counted as the total number of hospital beds, and the number of beds varied from 31 to 1517 in the hospitals. The average bed number was maintained at a similar level until 2006 and increased by an average of 5.13% over the last three years. Doctors and nurses were counted by the number of full-time professionals. The average number of doctors and nurses gradually increased over time, and the growth rates were about 55% and 50%,respectively, over the nine years. Including these labor-related inputs in the DEA model made efficiency analysis more parsimonious [41]. Inpatient days added both Medicare and Medicaid cases, and outpatient visits counted all visits to hospital emergency and outpatient facilities. Surgical operations that required resource-intensive work included the total number of inpatient and outpatient surgeries. The increase in the average number of outpatient visits was much higher than the increases in both output inpatient days and surgical operations.

Market competition as the explanatory variable was measured by the Herfindahl-Hirschman Index (HHI) as a widely accepted measure of market concentration. It was calculated by the sum of the squared market share of all hospitals in the market. A hospital’s market share is measured by its share of admissions across a hospital referral region. The U.S. Department of Justice [42] noted that a market concentration is categorized as either unconcentrated (<1500), moderately concentrated (1500 < HHI < 2500), and highly concentrated (>2500). If HHI is higher, market competition is lower. Over the nine years, the HHI scores gradually increased in Pennsylvania, ranging from 0.253 to 0.283, resulting in a relatively less competitive market.

This study controlled for four variables that were often discussed as driving factors in hospital productivity [3]. Hospital ownership was coded by whether hospitals held nonprofit status (coded 1) or for-profit (coded 2). Hospital ownership had mixed results. Studies claimed by property rights theory argued that for-profit hospitals performed more efficiently than nonprofit hospitals [43,44], while nonprofit hospitals were more productive than for-profits [13,45]. Guerrini et al. [44] observed that the efficiency measured by the productivity and cost savings of private health systems was better than those of public health systems. Other studies did not find a difference in productivity across different ownership types [46].

Competition for hospital services is dominated by location, which impacts hospital efficiency. The type of location was categorized by either urban (coded 1) or rural (coded 2). Hospitals often compete to secure provider network contracts, so health management originations’ (HMO) contracts influence hospital efficiency. Teaching hospitals are responsible for conducting medical research and for training the health care workforce so that productivity is lower in teaching hospitals (coded 1) than in non-teaching hospitals (coded 0) [37]. Each case of Medicare discharge and Medicaid discharge was a ratio of the number of each discharge out of the total number of discharges.

Table 2 summarizes the descriptive statistics of the variables. The efficiency scores over the nine years ranged between 0.378 and 1, and the mean score was 0.90. Among all discharges, the percentages of Medicare and Medicaid discharges were 52.91 and 16.20, respectively. The mean HHI is 0.266. For hospital competitiveness, 189 hospitals (26.92%) were located in less competitive areas (HHI > 0.25), 81 hospitals (11.54%) were in moderately competitive areas (0.15 < HHI < 0.25), and 432 hospitals (61.5%) were located in highly competitive areas (HHI < 0.15). Concerning the ownership of hospitals, 93.02 percent of the hospitals were nonprofit and 6.98 percent of them were for-profit. For the location of hospitals, 512 hospitals (72.93%) were located in urban areas and 190 hospitals (27.07%) were in rural areas, and the number of urban hospitals slightly decreased over time. A hospital on average had 7.17 contracts with HMOs. Teaching hospitals represented 55.70 percent and 44.30 percent were non-teaching hospitals.

## 3. Results

The bias-corrected TFP indices presented no steady productivity growth trend during the 2001–2009 period, but improved in general, except for the 2003–2004 and 2007–2008 periods (see Appendix A). On average, the TFP score was 1.05 that ranged from 0.942 to 1.166, and the number of hospitals over 1 was about 37. Since the lower mean scores were even close to 1, the results indicated that overall hospital productivity in Pennsylvania performed well during these periods. Unlike the stable average scores of the TFP indices, there was a broad spectrum of productive cases across the observed periods. As presented in Table 3, the number of efficient hospitals was quite varied, from 26 to 42. The low number of efficient hospitals was aligned with the low average productivity scores in 2001–2002, 2004–2005, and 2007–2008. The highest average growth rate occurred in the 2008–2009 period. The highest average growth rate was made in the 2008–2009 period. Sizeable variations in TFP scores across hospitals were observed in each period. In the 2008–2009 period, for example, the lowest and the highest productivity scores were 0.511 and 2.845, respectively.

No hospitals had efficient improvement in delivering health care services throughout the entire period. DMU 31 made record improvement six-times, except for 2004–2005 and 2007–2008, in which the average TFP scores were also at the below efficient level. A noticeable result was that a considerable number of hospitals experienced significant efficiency changes between two consecutive years in either upward or downward directions. Their productivity scores fluctuated overtime. DMU 64’s TFP score in the 2003–2004 term moved up from 0.34 to 2.529, and in the 2004–2005 term that increased seven times more.

There were only minor differences in the mean TFP scores between categories regarding types of location and ownership. Hospitals located in urban areas averaged 1.055, while rural hospitals’ mean score was 1.037. It could not be confirmed that urban hospitals performed meaningfully better than rural hospitals. The mean productivity score of for-profit hospitals (1.087) was slightly higher than its counterpart’s mean score (1.048), which was different from other studies [13]. This could be explained by the fact that private management prioritized service efficiency more than non-profit and public management hospitals under market competition [47].

The bootstrapped panel Tobit analysis results were that market competition had a statistically significant impact at the 10% level on hospital efficiency in delivering health care services, as presented in Table 4. The positive effect of market competition indicated that hospitals in less competitive areas run more efficiently than those located in more competitive areas. The intensity of market competition did not guarantee improvement in hospital efficiency in Pennsylvania. In the health care market, competition has generally influenced service provision and managerial practices, but its influences may vary in terms of efficiency in health care services. High competition offered more service provisions that eventually resulted in increased costs and decreased efficiency under cost-based reimbursement [20].

For-profit hospital ownership was positively significant at the 5% level, which indicated that for-profit hospitals were technically more efficient than nonprofit hospitals. Studies reported mixed results [2,3,6,14,48]. In this study, for-profit hospitals in Pennsylvania were more responsible in the use of resources and services that efficiently maximized profits. This study found that urban hospitals were negatively associated with hospital efficiency but not at a significant level. In Pennsylvania, rural hospitals were under more severe financial pressures than urban hospitals [49]. The share of rural hospitals operating around the break-even point was 6.0%, and the percent of rural hospitals operating under negative margins was 43.3% in 2010 [50].

The demand for HMOs tended to be high when hospital inefficiency was great [3]. Although there was no statistical significance, teaching hospitals indicated a positive effect on efficiency. Leleu et al. [14] also found that teaching hospitals were more efficient than their counterpart hospitals in accounting for quality because of the U.S. Hospital Readmission Reduction Program’s penalties. Regarding payment policies, the percentage of Medicare discharges had no significant impacts on hospital efficiency. Nevertheless, a higher the percentage of Medicaid discharge (*p* < 0.10) at the hospital negatively affected hospital efficiency, which could be explained by lower Medicaid reimbursement rates than Medicare cases.

## 4. Discussion and Conclusions

The results of hospitals’ performance growth in Pennsylvania trended in positive directions even though the growth remained relatively unchanged from 2001 to 2009. The hospitals’ average total factor productivity scores presented performance improvement in the five periods, including about 55 percent of the hospitals per year. The magnitude of underperforming hospitals’ total factor productivity scores was smaller across the hospitals. A merger between hospital systems to ensure access to timely and effective health care for residents and communities in Pennsylvania could lead to productivity growth in the hospitals over time. This study also observed some variations in total factor productivity scores across the hospitals. Thus, hospitals that lag behind in productivity growth should carefully benchmark productive peers to find out better strategies for their health care services.

Hospital services are often expected to behave differently when the market is competitive in order to increase efficiency. However, contradictory conclusions have been provided. This study confirmed that hospitals in lower competitive market areas were more efficient than hospitals in highly competitive market regions [51]. Hospitals in highly competitive regions tended to invest more inputs than its counterpart hospitals [6], but higher inputs did not bring high return proportionally in the case of Pennsylvania. As this study found a negative impact of market competition on hospital efficiency, market competition is therefore not always a perfect solution for operating hospitals efficiently when delivering health care services. A practical implication is that efficiency improvement should be aligned with resource management to balance between inputs and outputs. Hospital administrators should look for various approaches to lower unnecessary input resources, so that hospital efficiency could be improved.

Since a higher proportion of Medicaid discharges negatively affected hospital efficiency, those hospitals with a higher proportion of Medicaid patients need to pay special attention to improving their efficiency. Furthermore, if this phenomenon is related to lower reimbursement rates from Pennsylvania’s Medicaid program, hospitals should re-negotiate with the state Medicaid agency about the Medicaid reimbursement rates. For-profit hospitals turned out to be more efficient than their nonprofit peers as they tended to provide a smaller number of services with higher profit margins. Administrators and stakeholders in nonprofit hospitals need to understand the inherent dilemmas that nonprofit hospitals encounter (e.g., service to low-income and uninsured patients, the provision of a wider array of hospital services).

This study used a balanced dataset to estimate Malmquist productivity scores so that hospitals that missed any single data point in input and output measures across the nine years were excluded. Although an imputation method was not applied to hold hospitals with missing values due to an outlier issue, a minimum requirement of homogeneity in the establishment of a peer group may be questioned. Another methodological concern is about the selection of inputs and outputs, which is a typical drawback of DEA-based techniques. This study thoroughly selected input and output measures based on the objectives of this study and the exclusivity and exhaustiveness criteria that were used in previous research [52] but it is not entirely free from lingering concerns about whether the input-output combination can capture all aspects of hospital performance. Future research may narrow the scope of hospital services when they explore output quality in specialized fields.

## Figures and Tables

**Table 1:** Descriptive summaries of efficiency measures.

Measures	2001	2002	2003	2004	2005	2006	2007	2008	2009
Input									
Beds	263 (191)	263 (191)	262 (196)	264 (200)	263 (207)	267 (212)	274 (220)	279 (230)	279 (234)
Doctors ^1^	38 (129)	38 (129)	38 (129)	41 (158)	43 (160)	45 (159)	47 (158)	56 (217)	59 (227)
Nurses ^2^	259 (272)	259 (272)	277 (294)	293 (347)	305 (360)	325 (382)	342 (407)	375 (431)	388 (442)
Output									
Inpatient days	67,374 (58,248)	67,647 (58,560)	68,492 (61,706)	69,059 (63,305)	70,060 (65,962)	70,959 (67,819)	72,803 (70,246)	72,638 (70,718)	70,944 (70,963)
Surgical operations	10,126 (7444)	9884 (7137)	9930 (7381)	10,129 (7576)	10,342 (8091)	10,717 (8700)	10,369 (8195)	10,300 (8141)	10,046 (8754)
Outpatient visits	227,376 (190,083)	227,772 (182,636)	232,176 (186,479)	237,178 (195,440)	256,522 (224,350)	266,509 (234,149)	275,636 (245,995)	284,825 (254,678)	286,115 (253,714)

Notes: ^1^ full time equivalent number of physicians and dentists; ^2^ full time equivalent number of registered nurses; all measures are counted; standard deviations in parentheses.

**Table 2:** Descriptive summaries of variables.

	Obs.	Mean	SD	Min.	Max.
Efficiency	702	0.900203	0.1168394	0.378017	1
HHI	702	0.265937	0.3432643	0.0006448	1
Nonprofit hospitals	693	93.02%	-	-	-
For-profit hospitals	49	6.98%	-	-	-
Hosptals in urban areas	512	72.93%	-	-	-
Hospitals in rural areas	190	27.07%	-	-	-
HMO contracts	702	7.173789	6.601863	0	48
Teaching hospitals	391	55.7%	-	-	-
Nonteaching hospitals	311	44.3%	-	-	-
Medicare discharge	702	0.5291807	0.1383379	0.0321762	1.128692
Medicaid discharge	702	0.1620326	0.0796902	0.0176154	0.5551134
HHI < 0.15	432	61.54%	-	-	-
0.15 < HHI < 0.25	81	11.54%	-	-	-
HHI > 0.25	189	26.92%	-	-	-

**Table 3:** Summaries of bootstrapped productivity changes of Malmquist indices.

	2001/ 2002	2002/ 2003	2003/ 2004	2004/ 2005	2005/ 2006	2006/ 2007	2007/ 2008	2008/ 2009	Average
Average	0.980	1.110	1.042	0.965	1.134	1.067	0.942	1.166	1.051
Urban	0.958	1.155	1.060	0.961	1.105	1.074	0.932	1.195	1.055
Rural	1.042	0.976	0.990	0.977	1.216	1.046	0.970	1.080	1.037
Nonprofit	0.982	1.096	1.043	0.974	1.125	1.080	0.925	1.160	1.048
For profit	0.953	1.311	1.024	0.833	1.260	0.870	1.191	1.253	1.087

**Table 4:** Results of Tobit regression analysis.

Variable	Coefficient	SE	z	*p* > z
HHI	0.0122009 *	0.0071232	1.71	0.087
Ownership	0.0137529 **	0.0062494	2.20	0.028
Location_urban	−0.0020146	0.0079633	−0.25	0.800
HMO contracts	0.0006286	0.0004444	1.41	0.157
Teaching status	0.0049216	0.0068671	0.72	0.474
% Medicare discharge	0.0012537	0.0177059	0.07	0.944
% Medicaid discharge	−0.0478249	0.0288012	−1.66	0.097
Constant	0.637066	0.0132213	48.18	0.000
Rho	1.25 × 10^−33^	4.82 × 10^−18^		
		Wald chi2 = 10.38		
Log likelihood	940.03575	Prob > chi2 = 0.1678		

Notes: * *p* < 0.10; ** *p* < 0.05; *** *p* < 0.01.

## Data Availability

Restrictions apply to the availability of these data. Data was obtained from the American Hospital Association and are available from the American Hospital Association with the permission of the American Hospital Association.

## Appendix A

**Table A1:** Bootstrapped productivity changes of Malmquist indices, 2001–2009.

Hospital ID	2001/ 2002	2002/ 2003	2003/ 2004	2004/ 2005	2005/ 2006	2006/ 2007	2007/ 2008	2008/ 2009
1	0.882	1.222	0.849	0.681	2.835	0.568	0.767	0.708
2	0.892	1.088	0.826	1.167	1.039	0.810	1.338	0.511
3	0.820	1.046	1.010	0.664	1.793	1.047	0.842	0.524
4	1.132	1.271	0.629	1.159	1.130	1.130	0.754	1.261
5	2.176	0.580	1.071	0.488	1.281	1.114	0.916	1.330
6	0.821	1.024	1.070	1.174	0.815	1.150	0.652	1.562
7	0.583	2.151	0.790	1.103	0.771	0.873	0.659	2.032
8	1.162	0.790	1.157	1.147	0.828	1.117	0.835	0.837
9	0.988	0.913	0.968	0.568	1.680	0.833	1.104	0.968
10	0.646	2.061	0.627	1.052	0.609	1.841	0.781	1.131
11	1.007	1.162	0.719	1.392	0.567	1.946	1.123	0.957
12	0.903	1.306	1.002	0.853	0.520	1.465	0.744	1.439
13	1.131	1.082	1.253	0.835	1.106	0.697	1.721	0.976
14	0.591	1.085	1.120	0.848	1.345	0.941	0.653	1.384
15	1.178	0.923	1.043	0.608	1.473	1.000	0.803	1.406
16	0.906	0.808	1.068	0.541	1.904	0.983	1.063	0.863
17	1.249	0.940	0.924	0.873	0.812	1.414	0.966	0.927
18	0.487	2.007	0.775	1.034	1.000	0.820	0.819	2.837
19	0.744	0.718	1.842	0.723	1.245	1.182	0.772	0.898
20	1.496	0.615	1.659	0.901	1.281	0.801	0.681	2.021
21	1.296	0.593	0.970	1.173	1.115	0.715	1.197	0.942
22	0.738	1.291	0.667	2.481	0.814	1.037	0.587	0.973
23	1.015	1.062	1.045	0.700	1.178	0.969	1.217	0.907
24	0.682	1.592	0.833	0.638	1.385	1.018	0.925	0.804
25	0.832	1.549	1.081	0.879	0.904	1.069	1.249	0.943
26	1.153	0.539	1.264	0.922	0.963	0.975	0.871	1.741
27	1.300	0.854	0.964	0.716	2.110	0.793	0.947	0.604
28	0.899	1.109	1.245	0.823	1.027	0.823	1.023	0.650
29	0.870	0.999	1.174	0.546	1.938	1.016	1.304	0.660
30	1.089	1.171	0.701	1.135	0.964	1.482	0.682	1.282
31	1.373	1.073	1.032	0.560	1.055	1.184	0.940	1.230
32	0.748	1.129	0.960	1.193	0.834	1.196	0.637	1.888
33	0.936	1.052	1.243	0.827	0.751	0.543	1.154	1.773
34	1.633	0.778	1.084	1.002	1.179	0.960	0.939	0.599
35	0.911	1.078	0.904	0.841	1.628	0.595	1.045	1.043
36	0.682	1.645	0.787	0.867	0.882	1.529	0.793	1.040
37	0.808	1.102	0.867	1.052	0.657	1.625	0.993	1.018
38	0.635	1.513	1.009	1.222	0.673	1.066	0.981	1.159
39	1.094	0.947	1.606	0.850	0.814	0.660	1.792	0.976
40	0.632	1.027	1.192	0.981	1.055	1.196	0.683	1.071
41	1.187	0.836	1.148	0.620	1.949	0.817	0.823	1.231
42	0.784	0.770	0.598	0.746	2.129	0.944	1.198	1.010
43	0.969	1.044	0.951	0.982	0.539	1.413	0.873	0.944
44	0.846	2.100	0.493	1.150	0.925	1.220	0.720	1.358
45	0.949	0.706	1.631	0.803	1.071	1.271	0.616	1.337
46	1.068	0.741	1.651	0.863	0.968	1.215	0.634	1.445
47	1.245	0.743	1.074	1.068	1.133	0.747	0.909	0.921
48	0.940	0.987	0.527	1.609	1.034	1.149	0.955	0.667
49	1.007	1.051	0.958	0.803	1.084	1.128	1.168	0.948
50	0.572	1.920	0.698	0.846	1.378	1.226	0.877	0.783
51	0.645	1.855	1.044	1.071	1.007	0.906	1.152	1.285
52	1.122	0.585	1.385	1.211	0.757	0.955	0.852	1.989
53	1.105	0.867	1.220	0.712	1.911	0.726	0.820	0.820
54	0.909	0.988	1.615	0.568	1.193	0.809	1.086	0.770
55	0.869	0.916	1.194	0.658	1.310	1.093	0.844	1.013
56	1.022	1.187	0.824	0.864	1.069	1.532	0.810	1.029
57	2.110	0.777	1.245	0.980	0.686	1.291	0.923	1.063
58	0.693	1.062	1.148	1.208	0.897	1.132	0.632	1.923
59	0.807	1.374	1.062	0.977	0.817	0.659	0.624	2.410
60	1.304	0.950	0.971	1.100	1.198	0.812	1.162	0.585
61	1.149	0.757	0.982	0.874	0.953	1.157	1.255	0.829
62	0.577	2.558	0.531	0.940	0.791	1.838	0.692	1.042
63	0.775	0.937	0.916	0.846	0.929	1.657	0.869	0.887
64	0.654	1.354	0.340	2.529	0.956	1.160	0.904	1.277
65	0.898	1.024	1.638	0.874	0.955	0.528	2.174	0.892
66	0.857	0.768	1.396	0.897	0.969	1.037	0.812	1.325
67	1.152	0.798	1.198	0.601	2.031	0.779	0.746	1.470
68	1.068	0.798	1.015	0.464	1.952	0.863	1.237	0.924
69	0.990	1.239	0.836	1.050	0.577	1.375	1.164	0.807
70	0.869	1.134	0.839	1.181	0.849	1.248	0.759	2.845
71	0.984	0.906	1.655	0.744	1.039	1.429	0.624	1.215
72	0.900	0.929	1.693	0.908	0.788	1.349	0.684	1.306
73	2.389	0.964	1.173	0.880	1.135	0.872	0.628	1.721
74	0.796	1.064	0.509	2.301	0.863	1.007	1.047	0.624
75	0.770	1.092	0.991	0.789	1.390	0.965	1.128	0.960
76	0.576	1.592	0.937	0.781	1.497	0.933	1.150	0.858
77	0.492	1.740	0.786	1.311	0.902	0.936	1.048	1.459
78	1.259	0.531	1.378	1.241	0.849	0.850	0.932	1.064
